# Investigating Biochemical and Developmental Dependencies of Lignification with a Click-Compatible Monolignol Analog in *Arabidopsis thaliana* Stems

**DOI:** 10.3389/fpls.2016.01309

**Published:** 2016-08-31

**Authors:** Jyotsna L. Pandey, Sarah N. Kiemle, Tom L. Richard, Yimin Zhu, Daniel J. Cosgrove, Charles T. Anderson

**Affiliations:** ^1^Department of Agricultural and Biological Engineering, The Pennsylvania State University, University ParkPA, USA; ^2^Center for Lignocellulose Structure and Formation, The Pennsylvania State University, University ParkPA, USA; ^3^Department of Biology, The Pennsylvania State University, University ParkPA, USA; ^4^Department of Chemistry, Altoona College, The Pennsylvania State University, AltoonaPA, USA

**Keywords:** lignin, click chemistry, monolignol analog, peroxidase, sodium azide, diphenylene iodonium, N-acetyl-L-cysteine

## Abstract

Lignin is a key structural component of plant cell walls that provides rigidity, strength, and resistance against microbial attacks. This hydrophobic polymer also serves a crucial role in water transport. Despite its abundance and essential functions, several aspects of lignin biosynthesis and deposition remain cryptic. Lignin precursors are known to be synthesized in the cytoplasm by complex biosynthetic pathways, after which they are transported to the apoplastic space, where they are polymerized via free radical coupling reactions into polymeric lignin. However, the lignin deposition process and the factors controlling it are unclear. In this study, the biochemical and developmental dependencies of lignification were investigated using a click-compatible monolignol analog, 3-*O*-propargylcaffeyl alcohol (3-OPC), which can incorporate into both *in vitro* polymerized lignin and *Arabidopsis thaliana* tissues. Fluorescence labeling of 3-OPC using click chemistry followed by confocal fluorescence microscopy enabled the detection and imaging of 3-OPC incorporation patterns. These patterns were consistent with endogenous lignification observed in different developmental stages of *Arabidopsis* stems. However, the concentration of supplied monolignols influenced where lignification occurred at the subcellular level, with low concentrations being deposited in cell corners and middle lamellae and high concentrations also being deposited in secondary walls. Experimental inhibition of multiple lignification factors confirmed that 3-OPC incorporation proceeds via a free radical coupling mechanism involving peroxidases/laccases and reactive oxygen species (ROS). Finally, the presence of peroxide-producing enzymes determined which cell walls lignified: adding exogenous peroxide and peroxidase caused cells that do not naturally lignify in *Arabidopsis* stems to lignify. In summary, 3-OPC accurately mimics natural lignification patterns in different developmental stages of *Arabidopsis* stems and allows for the dissection of key biochemical and enzymatic factors controlling lignification.

## Introduction

Lignin, the second most abundant biopolymer on earth, is a complex and hydrophobic polyphenolic substance found in plant cell walls. Despite its abundance, lignin polymerization and deposition in plant cell walls (lignification) are poorly understood (Boerjan et al., 2003). Lignin provides structural integrity to plants (Chabannes et al., 2001; Jones et al., 2001; Boerjan et al., 2003), aids in pathogen defense (Cano-Delgado et al., 2003; Tronchet et al., 2010), and serves as a hydrophobic waterproofing agent facilitating vascular water transport (Boerjan et al., 2003; Weng and Chapple, 2010). The three most prevalent monomeric precursors of lignin (monolignols) are coniferyl alcohol (CA; which forms guaiacyl or G-lignin), sinapyl alcohol (which forms syringyl or S-lignin), and *p-*coumaryl alcohol (which forms hydroxyphenyl or H-lignin), but in addition to these, several other phenolic monomers can be incorporated into lignin (Boerjan et al., 2003; Ralph et al., 2004; Shadle et al., 2007; Lu and Ralph, 2008; Weng and Chapple, 2010), including synthetic monolignol analogs (Tobimatsu et al., 2013, 2014; Bukowski et al., 2014; Pandey et al., 2015). Unlike most biopolymer assembly processes, lignin polymerization is thought to be a chemically controlled process rather than biologically controlled, resulting in highly flexible monomer incorporation (Vanholme et al., 2010; Weng and Chapple, 2010). The complex structures of lignin polymers result from the way monolignols polymerize via undirected free radical coupling, leading to a multitude of inter-subunit linkages that connect phenylpropane units, including arylglycerol-β-aryl ether structures, phenylcoumaran structures, and bi-phenyl structures (Kirk and Farrell, 1987; Lewis and Yamamoto, 1990). Due to its structural heterogeneity and ether linkages, lignin is difficult to enzymatically degrade, and by masking other cell wall components it can inhibit the conversion of cellulose and hemicellulose into biofuels and other bio-products (Albersheim et al., 2010). Deeper understanding of lignin deposition should enable more efficient conversion of lignocellulosic biomass into high-value products and commodities.

Biosynthesis of monolignols from the amino acid phenylalanine proceeds in the cytoplasm via a series of enzymatic conversions (Zhao, 2016). Although the mechanism by which these monolignols are transported from the cytoplasm to the cell wall remains debated (Liu, 2012; Wang et al., 2013), they nonetheless move to the apoplastic space and are oxidized by peroxidases and laccases before undergoing a series of non-enzymatic free radical coupling reactions to form lignin polymers (Boerjan et al., 2003). At the subcellular scale, deposited lignin first appears at cell corners and the middle lamellae adhering adjacent plant cells, then appears progressively in the cell wall (Wardrop, 1957; Saka and Thomas, 1982; Terashima et al., 1988; Donaldson, 1991, 1992, 2001; Boerjan et al., 2003). Lignification is hypothesized to initiate at specific locations called nucleation sites, which serve as attachment sites for monolignols. Proposed nucleation sites include ferulate residues in the polysaccharides of grass cell walls, aromatic residues of structural proteins such as hydroxyproline-rich glycoproteins, and pectins (Keller et al., 1989; Ralph et al., 1995; Carpin et al., 2001; Grabber and Hatfield, 2005). However, at present scant evidence supports these hypotheses, and the molecular nature and nanoscale distribution of these nucleation sites are currently undefined. Another factor that can dictate where lignification occurs is the localization of oxidizing enzymes like peroxidases and laccases, as well as NADPH oxidases that produce hydrogen peroxide, the substrate of peroxidases (Boerjan et al., 2003; Lee et al., 2013; Schuetz et al., 2014).

Exogenously supplied monolignols with attached fluorophores can incorporate into plant tissues (Tobimatsu et al., 2011, 2013). However, these fluorophore-tagged monolignols are significantly larger than natural monolignols, and this may limit penetration through wall microstructures in different cell types and tissues, affecting incorporation patterns. Monolignol analogs with click chemistry-compatible tags are much closer in size to natural monolignols, and have recently been shown to incorporate into *in vitro*-synthesized lignin, as well as *Arabidopsis thaliana* (*Arabidopsis*) tissues including root epidermal cells and naturally lignifying tissues in the stem (Bukowski et al., 2014; Tobimatsu et al., 2014; Pandey et al., 2015). In the present work, the monolignol analog 3-*O*-propargylcaffeyl alcohol (3-OPC; Bukowski et al., 2014) was used to investigate the biochemical and developmental dependencies of lignification. 3-OPC contains a small alkyne modification compared to CA, allowing it to mimic the behavior and polymerization properties of the natural lignin monomer (Bukowski et al., 2014). Once incorporated into plant tissues, the degree and patterning of 3-OPC deposition can be detected using fluorescence microcopy after click-chemistry-enabled labeling with a fluorescent dye, providing a specific probe for new lignification.

In this study, 3-OPC incorporation in different developmental stages of *Arabidopsis* stems revealed that artificial lignification with this monolignol analog follows the same patterns as natural lignification. The effects of monolignol concentration on lignification patterns were investigated, revealing an apparent concentration-dependent localization that has relevance for understanding the natural lignification process. The involvement of native enzymes in the incorporation of 3-OPC was also investigated by testing the incorporation of 3-OPC in the presence of both peroxidase and peroxidase/laccase inhibitors. Incorporation of 3-OPC was also performed in the presence of other inhibitors of lignification, as well as exogenous peroxidases and hydrogen peroxide, to understand the effects of enzyme activity and availability on lignification in more detail than previously possible. This research highlights the utility of applying click-compatible monolignols to study the molecular dependencies of lignification, and can serve as a foundation for analyzing other unknown intricacies of lignin deposition, such as the molecular identities and distribution of lignin nucleation sites, as well as differential deposition of G-, S-, and H-lignin in different ultrastructural regions (Terashima et al., 1988; Fukushima and Terashima, 1990).

## Materials and Methods

### Reagents and Chemicals

Coniferyl alcohol, Horseradish peroxidase (HRP; type II, 150–250 units/mg), diphenylene iodonium (DPI), sodium azide, and *N*-acetyl-L-cysteine (NAC) were from Sigma. Alexa 594-azide, Alexa 488-azide, and Shandon^TM^ Cryomatrix^TM^ resin were from Thermo Scientific; monohydrate [2-(*N*-Morpholino)ethanesulfonic acid, MES] was from Research Organics; Murashige and Skoog salts were from Caisson Labs. 3-OPC was synthesized as described previously (Bukowski et al., 2014). Other commercial chemicals, including solvents, were from Sigma.

### Incorporation and Labeling of *Arabidopsis* Stem Sections

For developmental dependency experiments, top, middle, and bottom portions of 6-week-old and middle and bottom portions of 8-week-old *Arabidopsis* Col-0 ecotype stems were frozen in Shandon^TM^ Cryomatrix^TM^ resin, cryosectioned into 40-μm-thick transverse sections using a Leica CM1950 cryostat, placed in water and washed 3X with 1 mL water. Sections from each growth stage were transferred to 1 mL aqueous solution of 20 μM 3-OPC and 20 μM CA, or to an aqueous solution of 0.1 mg/mL HRP containing 20 μM 3-OPC and 20 μM CA. Sections were incubated at 25°C for 3 h with gentle rocking. After incorporation, sections were washed 4X with 1 mL water, transferred to 1 mL of click-labeling solution containing 1 mM ascorbic acid, 1 mM CuSO_4_, and 0.5 μM Alexa 594-azide in liquid MS medium (2.2 g/L Murashige and Skoog salts, 0.6 g/L MES, pH 5.6) and rocked at 25°C in the dark for 1 h. Sections were then washed 2X with 1 mL water, transferred to 1 mL of 96% ethanol, and rocked for 1 h to remove unbound monomers and dyes before washing 4X with 1 mL water.

For experiments testing different monolignol concentrations, bottom portions of 6-week-old *Arabidopsis* stems were cryosectioned as described above and placed in water. Sections were washed 3X with 1 mL water and transferred to 1 mL aqueous solutions of 0.05, 0.1, 0.2, 1, 5, 10, and 20 μM 3-OPC. Control sections were added to 1 mL aqueous solutions of 20 μM CA. These sections were incubated at 25°C for 3 h with gentle rocking. After incorporation, sections were washed 4X with 1 mL water, transferred to 1 mL of click-labeling solution containing 1 mM ascorbic acid, 1 mM CuSO_4_, and 0.5 μM Alexa 594-azide in liquid MS medium, and rocked at 25°C in the dark for 1 h. Sections were then washed 2X with 1 mL water, transferred to 1 mL of 96% ethanol, and rocked for 1 h to remove unbound monomers and dyes before washing 4X with 1 mL water.

To co-visualize cell walls and new lignification sites together, sections from bottom portions of 6-week-old *Arabidopsis* stems incubated with 0.05, 0.1, 0.2, and 10 μM 3-OPC were click-labeled as above, but with 0.5 μM Alexa 488-azide instead of Alexa 594-azide. The sections were then washed 3X with 1 mL water and labeled with 10 μM propidium iodide (PI) for 30 min. Sections were then washed 2X with 1 mL water, transferred to 1 mL of 96% ethanol, and rocked for 1 h to remove unbound monomers and dyes before washing 4X with 1 mL water.

For experiments analyzing how the duration of 3-OPC incorporation affects lignification patterns, sections of bottom portions of 6-week-old *Arabidopsis* stems were incubated with 5 μM 3-OPC and 15 μM CA for 10 min, 20 min, 30 min, 1 h, 2 h, 4 h, 8 h, 12 h, and 24 h. Control sections were treated with 20 μM CA for 24 h. Sections were then washed and click labeled with Alexa 594-azide as above. Short incorporation durations of 10 min were also tested with 3-OPC concentrations of 0.1 and 0.2 μM, followed by the same washing and click-labeling steps.

To test the effects of exogenous hydrogen peroxide and peroxidase, 40 μm sections of bottom portions of 6-week-old *Arabidopsis* stems were placed in water immediately after cryo-sectioning. Sections were then washed 3X with 1 mL water and transferred to 1 mL aqueous solutions of 20 μM 3-OPC and 20 μM CA containing 0.1 mg/mL HRP and 0.001% (v/v) hydrogen peroxide. Other sections were transferred to 1 mL aqueous solutions of 20 μM 3-OPC and 20 μM CA containing only 0.1 mg/mL HRP (no hydrogen peroxide). A third sub-set of sections was transferred to 1 mL aqueous solutions of 20 μM 3-OPC and 20 μM CA containing no HRP or hydrogen peroxide. These sections were incubated at 25°C for 3 h with gentle rocking. After incorporation, the sections were washed and click-labeled with 0.5 μM Alexa 594-azide as described above.

To test the effects of enzyme inhibitors, 40 μm sections of bottom portions of 6-week-old *Arabidopsis* stems were placed in water immediately after cryo-sectioning. Sections were then washed 3X with 1 mL water followed by treatments with three inhibitors: DPI, an NADPH oxidase inhibitor; NAC, an antioxidant and reactive oxygen species (ROS) scavenger; and sodium azide (NaN_3_), a peroxidase and laccase inhibitor. For testing the effects of inhibitors during incorporation, sections were incubated in 1 mL aqueous solution of 20 μM 3-OPC and 20 μM CA containing 15 μM DPI, 1 mM NAC, 1 mM NaN_3_, or no inhibitor for 3 h. For testing effect of inhibitors before incorporation, sections were transferred to 1 mL aqueous solutions of 15 μM DPI, 1 mM NAC, 1 mM NaN_3_, or no inhibitor for 1 h, washed 4x with 1 mL water, and incubated in 1 mL aqueous solutions of 20 μM 3-OPC and 20 μM CA for 3 h. After incorporation, sections were washed and click-labeled with 0.5 μM Alexa 594-azide as described above.

### Imaging of Stem Sections

Images of labeled and control stem sections were collected on a Zeiss Cell Observer SD spinning disk fluorescence confocal microscope using a 561 nm excitation laser with a 617/73 emission filter to image click-associated Alexa 594 fluorescence and PI labeling, a 488 nm excitation laser with a 525/50 emission filter to image click-associated Alexa 488 fluorescence, and a 405 nm excitation laser with a 450/50 emission filter to image autofluorescence associated with lignin. For imaging, 10× 0.5 NA and 20× 0.5 NA air immersion objectives, and a 63× 1.4 NA oil immersion objective, were used. Maximum projections of z series were generated using ImageJ, adjusting brightness for all images to the same minimum and maximum values to enable comparison of relative fluorescence intensities. Fluorescence intensities were quantified as raw integrated intensities per unit area using ImageJ after applying a uniform threshold to select lignified regions (Supplementary Figure S1), with the exception of images for inhibitor experiments, which due to widely varying fluorescence levels were auto-thresholded to standardize thresholded areas. Separate threshold values were set for images acquired under 405 and 561 nm channels.

## Results

The monolignol probe 3-OPC has been shown to incorporate into *in vitro-*polymerized lignin, also called dehydrogenation polymers (DHPs), as well as plant tissues using click chemistry-assisted labeling and fluorescence imaging (Bukowski et al., 2014). In this study, 3-OPC incorporation patterns were studied in *Arabidopsis* stem sections with an aim to probe the developmental and biochemical dependencies of the lignification process.

### Incorporation of 3-OPC in Different Developmental Stages of *Arabidopsis* Stems Occurs Primarily in Actively Lignifying Tissues

**FIGURE 1:**
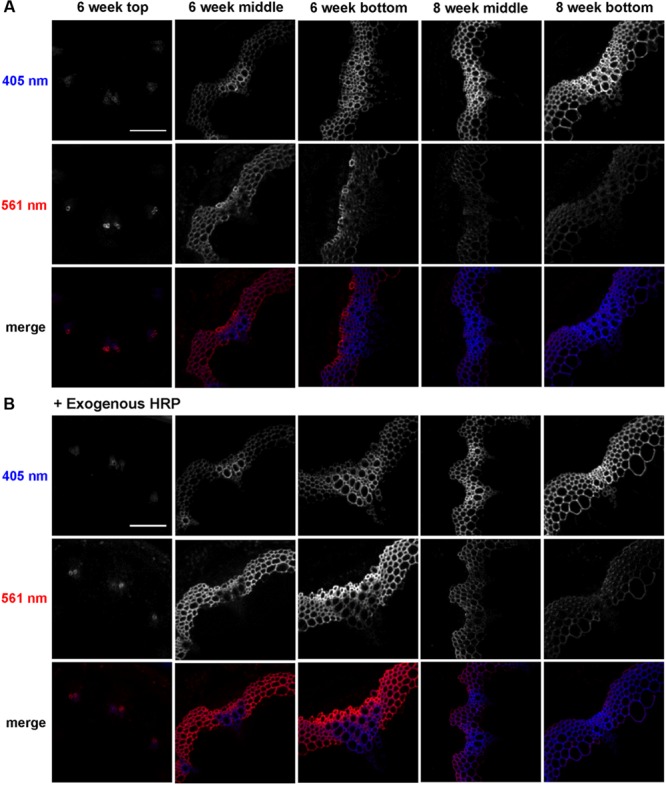
**Incorporation of 3-OPC in different developmental stages of *Arabidopsis* stems.** Autofluorescence (405 nm excitation) and click labeling (561 nm excitation) in sections of top, middle and bottom portions of 6-week-old and middle and bottom portions of 8-week-old *Arabidopsis* stems treated with **(A)** 20 μM 3-OPC and 20 μM CA without exogenous HRP for 3 h or **(B)** 20 μM 3-OPC and 20 μM CA with exogenous HRP for 3 h, labeled with Alexa 594-azide for 1 h, and washed with 96% ethanol for 1 h. The colored images on the bottom rows for each panel are merges of the 405 nm (blue) and 561 nm (red) channels. Images are contrast-enhanced maximum intensity projections of z series collected using a 20× objective with a 561 nm laser at 5% power and a 405 nm laser at 100% power, with 150 gain and 400 ms exposure time (scale bar, 100 μm). Three replicate experiments with at least three sections per experiment were imaged for each developmental stage.

To analyze developmental patterns of lignification, 3-OPC incorporation was studied in developing *Arabidopsis* inflore scence stems. Cryosections from top, middle, and bottom portions (youngest to oldest tissues) of 6-week-old and middle and bottom portions of 8-week-old stems were treated with 20 μM 3-OPC and 20 μM CA for 3 h, click-labeled with Alexa 594-azide for 1 h, washed to remove unbound monolignols and dye, and imaged using spinning disk confocal microscopy. We previously found (Bukowski et al., 2014) that 405 nm autofluorescence is mainly contributed by pre-existing lignins, and click-associated fluorescence is mainly contributed by newly incorporated lignins. **Figure 1A** shows different 3-OPC incorporation patterns in stems from different developmental stages. In sections from the top (younger) portions of 6-week-old stems, 405 nm autofluorescence shows that vascular bundles had only started to develop and lignify, and interfascicular fibers (IFFs) were not yet lignified (**Figure 1A**). Click labeling in these sections was limited to developing xylem vessels, and was evident in only a few cells. In sections from middle portions of 6-week-old stems, vascular bundles were more developed and lignified compared to the younger top portion, and IFFs were also starting to lignify, but still showed only faint autofluorescence. Click labeling was very strong in these sections, with much higher fluorescence intensities in IFFs than in vascular bundles (**Figure 1A**). In 6-week-old bottom sections, the most mature part of the 6-week-old stem, significant existing lignification was present in both IFFs and vascular bundles, with higher autofluorescence in the vasculature. Click labeling in these sections was strongest in xylary fibers, followed by IFFs, and negligible in vascular bundles, which showed very high 405 nm autofluorescence (**Figure 1A**). Click-labeling intensity in the IFFs of bottom sections was lower than in middle sections. Sections from middle portions of 8-week-old stems showed much higher autofluorescence than 6-week-old stems, with IFFs also being strongly lignified. Click labeling was lower but still detectable in these sections, with higher intensities in IFFs than in vascular bundles (**Figure 1A**; Supplementary Figure S2). In sections from bottoms of 8-week-old stems, very little click labeling was observed. Autofluorescence in these sections showed well lignified IFFs and vascular bundles (**Figure 1A**).

A similar experiment was performed to test 3-OPC incorporation in different developmental stages of *Arabidopsis* stems with addition of exogenous HRP, which can generate free radicals that drive lignin polymerization (Lee et al., 2013). Cryosections of top, middle and bottom portions of 6-week-old and middle and bottom portions of 8-week-old *Arabidopsis* stems were treated with 3-OPC and CA plus 0.1 mg/mL HRP, click-labeled, and imaged. Incorporation patterns observed in these samples (**Figure 1B**) were similar to those observed in **Figure 1A**, but total click-associated fluorescence increased. Addition of exogenous HRP alone did not result in lignification of non-lignifying tissues, but incorporation in naturally lignifying tissues was enhanced.

A quantification analysis (Supplementary Figure S2) of autofluorescence (405 nm) and click-labeling-associated fluorescence (561 nm) for 3-OPC incorporation experiments with and without exogenous HRP revealed that autofluorescence per unit area (Supplementary Figure S2A) increased from top to bottom in 6-week-old sections and further increased in 8-week-old middle and bottom sections. Supplementary Figure S2B shows the thresholded area selected for quantification (see Supplementary Figure S1 for procedure for selecting lignified area). Lignified area also increased from top to bottom in 6-week-old stems as lignified cells developed, becoming thicker and more lignified (**Figure 1**). The middle sections of 8-week-old stems showed slightly lower total lignified areas than 6-week-old bottom sections, because the diameters of these sections were smaller even though the cell walls were thicker. Eight-week-old bottom sections showed the largest lignified area as well the highest autofluorescence per unit area. Autofluorescence and lignified area were not greatly affected by the presence of exogenous HRP (Supplementary Figure S2). Click labeling in sections where 3-OPC was incorporated without exogenous HRP was highest per unit area in 6-week-old middle sections. Intensity per unit area increased from top to middle and decreased from middle to bottom sections. Click labeling then again decreased further from 6-week-old bottom to 8-week-old middle and bottom sections, with lowest intensities in 8-week-old bottom sections.

On adding exogenous HRP, click labeling was higher for all developmental stages except 6-week-old top sections (**Figure 1B**). Incorporation of 3-OPC was enhanced most significantly in 6-week-old bottom sections by HRP addition. With HRP, 6-week-old bottom sections showed the highest click labeling per unit area. The selected lignified area, based on autofluorescence, was largest in 6-week-old bottom sections, in both absence and presence of exogenous HRP (Supplementary Figure S2B).

### Changing 3-OPC Concentrations Alters Subcellular Patterns of Lignification in *Arabidopsis* Stem Sections

**FIGURE 2:**
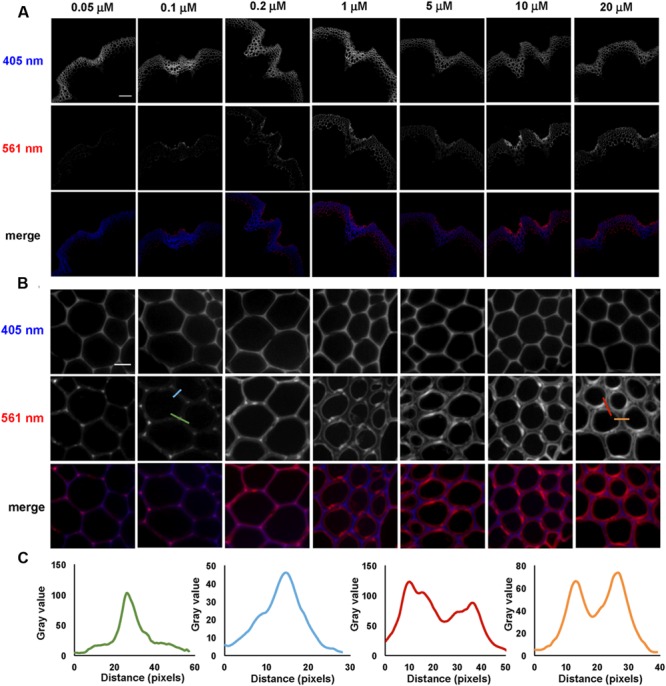
**Incorporation of 3-OPC in sections of bottom portions of 6-week-old *Arabidopsis* stems at different concentrations. (A)** Autofluorescence (405 nm excitation) and click labeling (561 nm excitation) in sections treated with CA as a control or 0.05, 0.1, 0.2, 1, 5, 10, or 20 μM 3-OPC for 3 h, labeled with Alexa 594-azide for 1 h, and washed with 96% ethanol for 1 h. **(B)** Higher magnification images of interfascicular fibers (IFFs) showing autofluorescence (405 nm excitation) and click labeling (561 nm excitation) in samples described above. **(C)** Fluorescence intensity profiles along the colored transect lines shown in **(B)**. Bottom rows for both **(A,B)** show merges of the 405 nm (blue) and 561 nm (red) channels. Images are contrast-enhanced maximum intensity projections of z series recorded using a 10× objective **(A)** and a 63× objective **(B)** with a 561 nm laser at 5% power and a 405 nm laser at 100% power, with gains of 150 for **(A)** and 50 for **(B)** and 400 ms exposure time. Different levels of brightness and contrast were used for **(B)** in order to clearly observe differences in incorporation patterns (scale bar, **A**: 100 μm; **B**: 10 μm). Three replicate experiments with at least three sections per experiment were imaged for each treatment.

Incorporation of 3-OPC at different concentrations was tested in sections from bottom portions of 6-week-old *Arabidopsis* stems. Sections were treated with 3-OPC at concentrations of 0.05, 0.1, 0.2, 1, 5, 10, and 20 μM, after which they were labeled with 0.5 μM Alexa 594-azide. **Figure 2** shows fluorescence images obtained for sections treated with different concentrations of 3-OPC. Click labeling increased with increasing 3-OPC concentrations (**Figure 2A**). Quantification (Supplementary Figure S3) revealed an increase in fluorescence per unit area as 3-OPC concentration increased from 0.05 to 10 μM, after which intensities did not increase, possibly due to saturation of the sections or a limiting click reagent. In these samples, the xylem in vascular bundles showed lower click labeling than xylary fibers and IFFs. Xylary fiber cells at the periphery of the vascular bundle, close to the phloem, showed stronger labeling than IFFs in all sections treated with 3-OPC. In IFF regions, cells in the middle of the bands showed less click labeling than cells on the peripheries neighboring the pith and cortex. Only naturally lignifying cells showed 3-OPC incorporation. **Figure 2B** shows incorporation patterns of 3-OPC in IFFs at higher magnification. At this scale, a difference in incorporation patterns of 3-OPC across differing concentrations is evident. In sections treated with low concentrations of 3-OPC, namely 0.05, 0.1, and 0.2 μM, incorporation occurred primarily in cell corners and middle lamellae, which are widely considered to be locations of lignification initiation (Saka and Thomas, 1982). In sections treated with higher concentrations of 3-OPC, namely 1, 5, 10, and 20 μM, incorporation at cell corners and middle lamellae was still detected, but labeling intensity was higher in secondary cell wall regions. **Figure 2C** shows intensity profiles across a cell corner (green) and cell wall (blue) for a section treated with 0.1 μM 3-OPC, indicating incorporation primarily in cell corners and middle lamellae (note differing *Y*-axis scales). Intensity profiles are also shown across a cell corner (red) and cell wall (orange) for a section treated with 20 μM 3-OPC, indicating higher fluorescence in cell wall regions. These data indicate that at low concentrations of 3-OPC, new lignin deposition occurs primarily at cell corners and middle lamellae, but at higher concentrations of 3-OPC, the secondary wall region becomes lignified to a higher degree than cell corners and middle lamellae.

**FIGURE 3:**
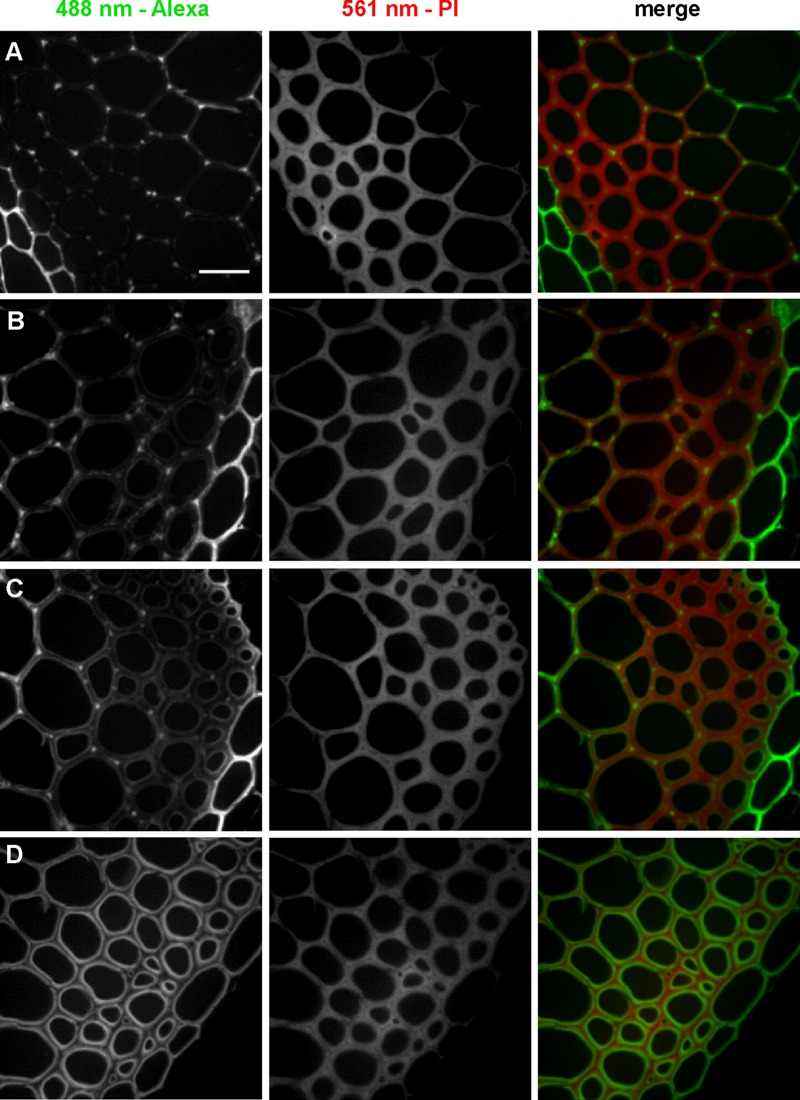
**Co-labeling of cell walls and incorporated 3-OPC in sections of 6-week-old *Arabidopsis* stem.** IFFs showing click labeling (488 nm excitation) and PI labeling (561 nm excitation) in 40 μm-thick sections of 6-week-old *Arabidopsis* stem sections treated with **(A)** 0.05 μM, **(B)** 0.1 μM, **(C)** 0.2 μM, and **(D)** 10 μM 3-OPC for 3 h, labeled with Alexa 488-azide for 1 h, labeled with PI for 30 min, and washed with 96% ethanol for 1 h. Images are contrast-enhanced maximum intensity projections of z series recorded using a 63× objective with a 488 nm laser at 5% power and a 561 nm laser at 5% power, with 50 gain and 400 ms exposure time. Colored images in the right column are merges of the 488 nm (green) and 561 nm (magenta) channels. Different ranges of brightness and contrast were used to reveal differences in incorporation patterns (scale bar, 20 μm). Three replicate experiments with at least three sections per experiment were imaged for each treatment.

To further investigate localization patterns of 3-OPC at different concentrations, co-labeling was performed with PI, which stains acidic polysaccharides, including de-methylated pectins, in cell walls (Rounds et al., 2011). Sections were treated with 0.05, 0.1, 0.2, or 10 μM 3-OPC for 3 h, after which they were labeled with 0.5 μM Alexa 488-azide, followed by labeling with PI for 30 min. **Figure 3** shows 3-OPC incorporation patterns relative to PI localization. A 488 nm excitation laser was used to detect click-associated fluorescence from Alexa 488-azide (left column, **Figure 3**) and a 561 nm excitation laser was used to detect regions of PI binding (middle column, **Figure 3**). The rightmost column shows a merged image for 561 nm (red) and 488 nm (green) channels. PI labeling was observed throughout cell walls, with less labeling in cell corners and middle lamellae, where significant incorporation of 3-OPC was observed at low concentrations. For sections treated with 10 μM 3-OPC, click labeling co-localized with PI labeling on inner edges of cell walls. These data further indicate at lower concentrations, 3-OPC is deposited at cell corners and middle lamellae, whereas at higher concentrations, 3-OPC accumulates more in secondary wall regions.

### Increasing Incubation Time Increases 3-OPC Accumulation, But Does Not Alter 3-OPC Deposition Patterns

**FIGURE 4:**
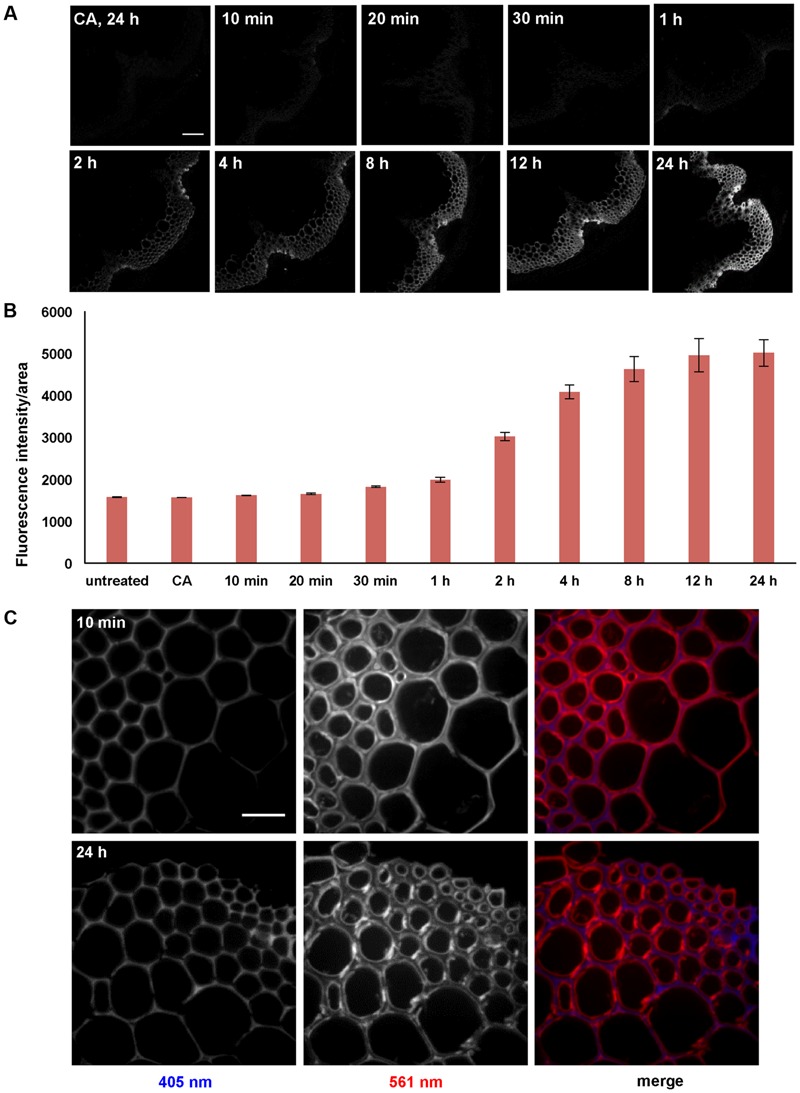
**Time-course of incorporation of 3-OPC in sections of 6-week-old *Arabidopsis* stems. (A)** Click labeling (561 nm excitation) in sections treated with 5 μM 3-OPC and 15 μM CA for 10 min, 20 min, 30 min, 1 h, 2 h, 4 h, 8 h, 12 h, and 24 h, labeled with Alexa 594-azide for 1 h, and washed with 96% ethanol for 1 h. Control sections treated with 20 μM CA for 24 h and labeled with Alexa 594-azide for 1 h (panel labeled CA, 24 h) were included. Images are contrast-enhanced maximum intensity projections of z series recorded using a 10× objective with a 561 nm laser at 5% power and a 405 nm laser at 100% power, with 500 gain and 400 ms exposure time (scale bar, 100 μm). **(B)** Quantification of fluorescence intensities per unit area for the above described samples. Sections not treated with monolignols or Alexa 594-azide were also included. Data were averaged from three replicate experiments with four sections imaged for each treatment; error bars indicate standard error. **(C)** Autofluorescence (405 nm excitation) and click labeling (561 nm excitation) in IFFs of 40 μm-thick sections of 6-week-old *Arabidopsis* stem treated with 5 μM 3-OPC and 15 μM CA for 10 min and 24 h, labeled with Alexa 594-azide for 1 h, and washed with 96% ethanol for 1 h. The colored images (right column) are merges of the 405 nm (blue) and 561 nm (red) channels. Images are contrast-enhanced maximum intensity projections of z series recorded with a spinning disk fluorescence confocal microscope recorded using a 63× objective with a 561 nm laser at 5% power and a 405 nm laser at 100% power, with 50 gain and 400 ms exposure time. Different ranges of brightness and contrast were used in order to observe any differences in incorporation patterns at 63× (scale bar, 20 μm).

A time-course experiment was performed with stem sections from 6-week-old *Arabidopsis* plants to investigate whether incorporation intensities or patterns change with incubation time. Sections were treated with 5 μM 3-OPC and 15 μM CA for 10 min, 20 min, 30 min, 1 h, 2 h, 4 h, 8 h, 12 h, and 24 h, and labeled with Alexa 594-azide. Click labeling was only observed in IFFs and vascular bundles, which are naturally lignifying tissues (**Figure 4A**). Click labeling per unit area increased with increasing incubation time, reaching a maximum intensity at 24 h. A steeper increase in labeling intensity from the 1 h incorporation time point to the 12 h time point was observed, after which there was only a slight increase over the next 12 h to the 24 h time point (**Figure 4B**). For all time points, the most intense click labeling was present in xylary fibers, followed by IFFs, with negligible click labeling in xylem vessels. Images at higher magnification were collected to investigate whether there were any differences in monolignol incorporation patterns over a time-course of 24 h. **Figure 4C** shows images of IFFs from sections treated with 5 μM 3-OPC and 15 μM CA for 10 min and 24 h. For the 10-min time point, labeling was more prominent in secondary wall regions of the IFFs, as observed in sections treated with 5 μM 3-OPC for 3 h (**Figure 2B**).

**FIGURE 5:**
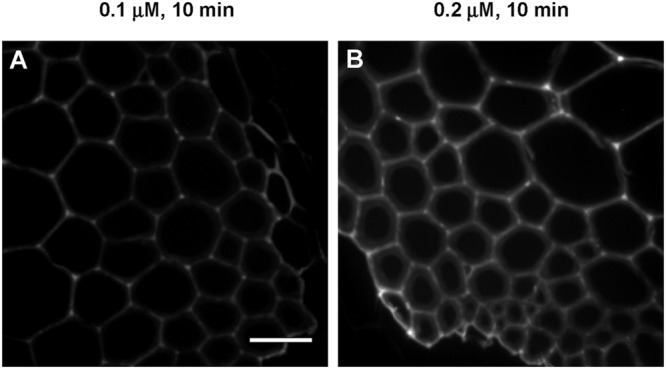
**Incorporation of 3-OPC in IFFs of sections of 6-week-old *Arabidopsis* stems at low concentrations and for short durations.** Click labeling-associated fluorescence (561 nm excitation) in sections treated with **(A)** 0.1 μM and **(B)** 0.2 μM 3-OPC for 10 min, labeled with Alexa 594-azide for 1 h, and washed with 96% ethanol for 1 h. Images are contrast-enhanced maximum intensity projections of z series recorded with a spinning disk fluorescence confocal microscope recorded using a 63× objective with a 561 nm laser at 5% power, with a gain of 50 and 400 ms exposure time (scale bar, 20 μm). Three replicate experiments with at least three sections per experiment were imaged for each treatment.

To better understand the contributions of incorporation duration and monolignol analog concentration on patterns of lignification, sections treated with low concentrations of 3-OPC for short periods of time were also imaged. **Figure 5** shows high magnification images of IFFs from sections treated with 0.1 μM and 0.2 μM 3-OPC for 10 min, labeled with Alexa 594-azide. For both samples, more click labeling was observed in cell corners and middle lamellae, indicating that even with a short incubation time, the same patterns of lignification occur at low concentrations of 3-OPC.

### Testing Biochemical Factors That Control Lignification in *Arabidopsis* Stems

From the data described above it was evident that the addition of HRP enhanced incorporation of 3-OPC in *Arabidopsis* stem sections, but only in tissues that normally lignify (**Figure 1**, Supplementary Figure S2). This implies that H_2_O_2_, which is required for the activities of HRP and endogenous peroxidases, might be supplied by endogenous enzymes that localize only in lignifying cells (Lee et al., 2013). Therefore, we hypothesized that the localization of lignification depends on the localization of certain peroxide-producing enzymes, and that if H_2_O_2_ were supplied along with HRP during 3-OPC incorporation, lignification would also occur in non-lignifying cell types. To test this hypothesis, sections of 6-week-old *Arabidopsis* stems were treated with 20 μM 3-OPC and 20 μM CA with added H_2_O_2_ (0.001%, v/v) and with or without exogenous HRP for 3 h, then labeled with Alexa 594-azide. As controls, sections treated with only 20 μM 3-OPC and 20 μM CA and without added H_2_O_2_ were also included.

**FIGURE 6:**
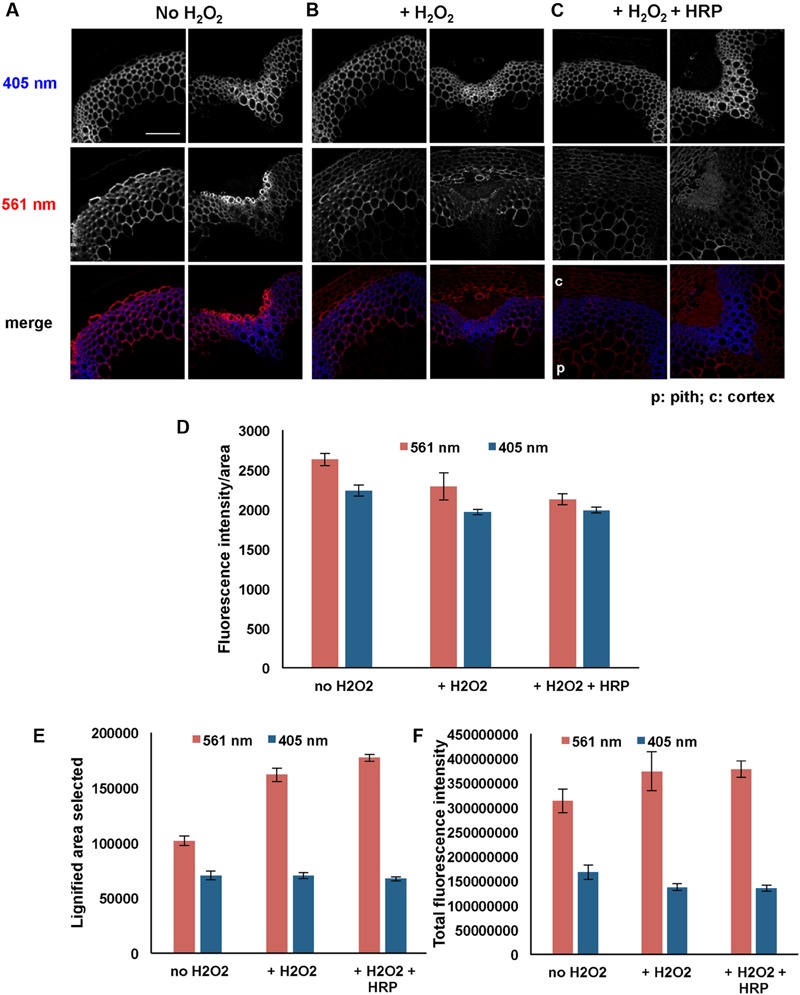
**Incorporation of 3-OPC in presence and absence of exogenous hydrogen peroxide (H_2_O_2_) in sections of 6-week-old *Arabidopsis* stems.** Top: autofluorescence (405 nm excitation) and click labeling (561 nm excitation) in sections treated with 20 μM 3-OPC and 20 μM CA in **(A)** absence of H_2_O_2_, **(B)** presence of 0.001% H_2_O_2_ and **(C)** presence of 0.001% H_2_O_2_ as well as exogenous HRP, for 3 h, labeled with Alexa 594-azide for 1 h, and washed with 96% ethanol for 1 h. c = cortex, p = pith. Colored images on the bottom row are merges of the 405 nm (blue) and 561 nm (red) channels. Images are contrast-enhanced maximum intensity projections of z series recorded using a 20× objective with a 561 nm laser at 5% power and a 405 nm laser at 100% power, with 150 gain and 400 ms exposure time (scale bar, 100 μm). **(D–F)** Quantification of **(D)** fluorescence intensities per unit area, **(E)** lignified (thresholded) area selected, and **(F)** total fluorescence intensity for click labeling-associated fluorescence (561 nm excitation, red) and autofluorescence (405 nm excitation, blue) in sample sections described above. Data were averaged from three replicate experiments with three sections images for each treatment; error bars indicate standard error.

**Figures 6A–C** shows images from these experiments. Sections treated with 3-OPC in the presence of added H_2_O_2_ (**Figure 6B**) displayed slightly lower fluorescence per unit area than sections treated with 3-OPC in the absence of added H_2_O_2_, although this difference was not statistically significant (**Figure 6D**, *p* > 0.05, *t*-test). However, in the presence of added H_2_O_2_, fluorescence was detected in walls of non-lignifying cells including cortex and pith (**Figure 6B**). Quantification revealed that selected click-labeled areas (**Figure 6E**) and total fluorescence intensities (**Figure 6F**) increased with added H_2_O_2_, although only the difference in labeled area was significant (*p* < 0.05, *t*-test). Lower fluorescence per unit area may be attributed to dilution of incorporated 3-OPC due to more cell wall area being available for incorporation in the presence of added H_2_O_2_. Sections treated with both H_2_O_2_ and HRP showed click labeling in both cortex and pith, as well as in naturally lignifying cell walls (**Figure 6C**). Fluorescence per unit area was significantly lower, but labeled area and total fluorescence were higher, for samples treated with both H_2_O_2_ and HRP than for untreated controls (**Figures 6D–F**, *p* < 0.05, *t*-test). There were no significant differences in fluorescence per unit area, lignified area, or total fluorescence between samples treated with H_2_O_2_ alone and samples treated with both H_2_O_2_ and HRP (**Figures 6D–F**, *p* > 0.05, *t*-test). Higher magnification images of IFFs and vascular bundles of these sections treated with 3-OPC in the presence of H_2_O_2_ were collected to observe incorporation patterns in cell walls (Supplementary Figure S4). In IFFs of both samples (Supplementary Figures S4A,C), click labeling was observed in the secondary cell wall region and not in cell corners and middle lamellae, as also observed for samples treated with 3-OPC without added H_2_O_2_ (**Figure 2B**, 20 μM). In vascular bundles of both samples, click labeling was mainly observed in xylary fibers and not xylem vessels, as also observed previously for samples treated with 3-OPC without added H_2_O_2_ (**Figure 2A**, 20 μM). Therefore, addition of H_2_O_2_ did not significantly alter the pattern of monolignol deposition within IFFs and vascular bundle cell walls. However, in samples treated with both H_2_O_2_ and HRP (Supplementary Figures S4C,D), concentrated spots of click labeling were observed outside the cell wall as well, possibly owing to 3-OPC polymerization in solution.

To investigate the functions of peroxidases and laccase enzymes in lignification as detected by 3-OPC incorporation, the effects of inhibitors of these enzymes on 3-OPC incorporation were tested. Inhibitors included DPI, an inhibitor of the NADPH oxidases (Li and Trush, 1998; Lee et al., 2013; Pesquet et al., 2013) that produce H_2_O_2_. H_2_O_2_ is required by peroxidases for their oxidizing activity that converts monolignols into free radicals. NAC is an antioxidant and ROS scavenger (Pesquet et al., 2013) that should inhibit free radical formation and hence 3-OPC incorporation into plant tissue. Sodium azide (NaN_3_) inhibits both peroxidases (Brill and Weinryb, 1967) and laccases (Johannes and Majcherczyk, 2000; Patel et al., 2014) and was used to test whether peroxidases and laccases are involved in 3-OPC incorporation. The three aforementioned inhibitors were tested in two different ways: they were applied before the incorporation step for 1 h, after which sections were washed and incorporation of 20 μM 3-OPC was performed for 3 h, or they were applied during the 3 h incorporation step along with 3-OPC. A control treatment, with 3-OPC only and no inhibitors, was also included.

**FIGURE 7:**
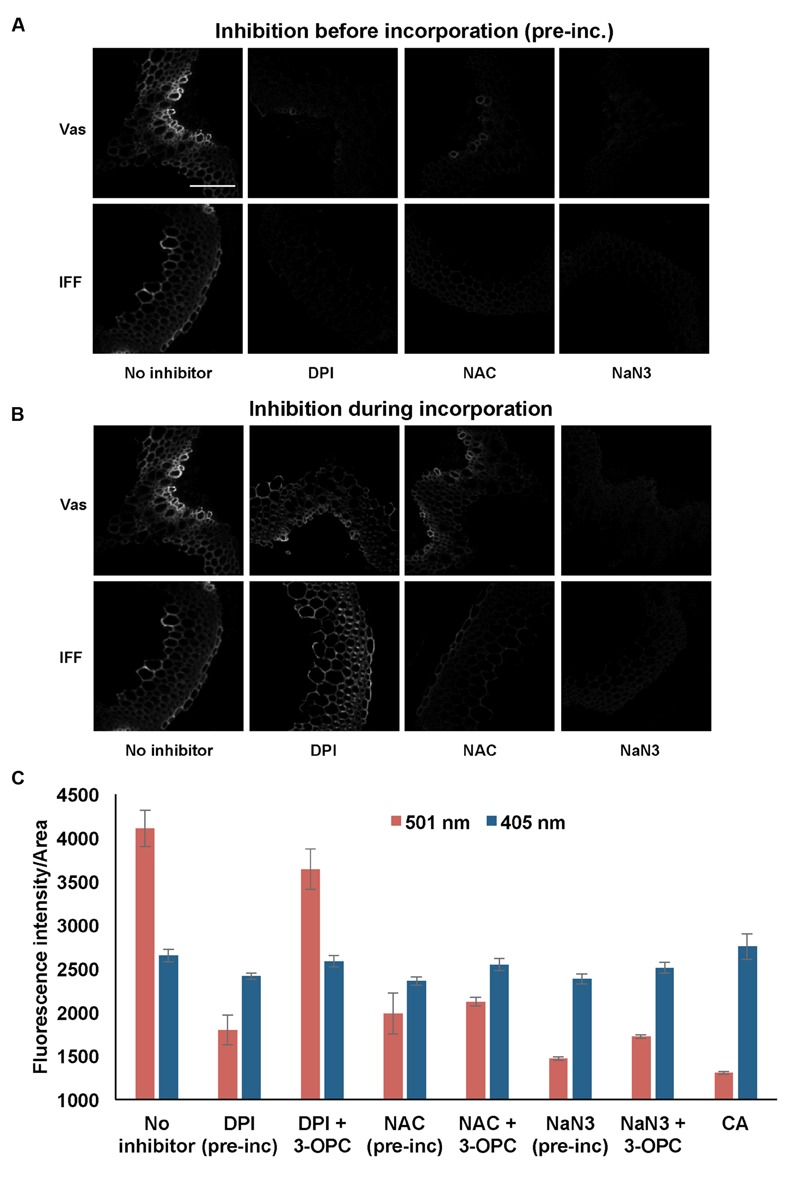
**Effect of inhibitors on incorporation of 3-OPC in sections of 6-week-old *Arabidopsis* stems.** Inhibitors tested include: 15 μM diphenylene iodonium (DPI), an NADPH oxidase inhibitor, 1 mM *N*-acetyl-L-cysteine (NAC), an antioxidant and ROS scavenger and 1 mM sodium azide (NaN3), a peroxidase inhibitor. **(A)** Click labeling (561 nm excitation) in sections treated with indicated inhibitors (or no inhibitor) for 1 h, followed by 20 μM 3-OPC and 20 μM CA for 3 h, then labeled with Alexa 594-azide for 1 h, and washed with 96% ethanol for 1 h. **(B)** Click labeling (561 nm excitation) in sections treated with 20 μM 3-OPC and 20 μM CA along with the indicated inhibitors (or no inhibitor) for 3 h, labeled with Alexa 594-azide for 1 h, and washed with 96% ethanol for 1 h. Both vascular bundles (Vas) and IFFs are shown for each treatment. Images are contrast-enhanced maximum intensity projections of z series recorded using a 20× objective with a 561 nm laser at 5% power, with 150 gain and 400 ms exposure time (scale bar, 100 μm). **(C)** Quantification of fluorescence intensities per unit area for click labeling-associated fluorescence (561 nm excitation, red) and autofluorescence (405 nm excitation, blue) in stem sections treated with different inhibitors before (pre-inc) and during incorporation with 3-OPC as described above. As negative controls, sections treated with 20 μM CA for 3 h were included. Data were averaged from three replicate experiments with four sections images for each treatment; error bars indicate standard error (pre-inc: pre-incubation).

**FIGURE 8:**
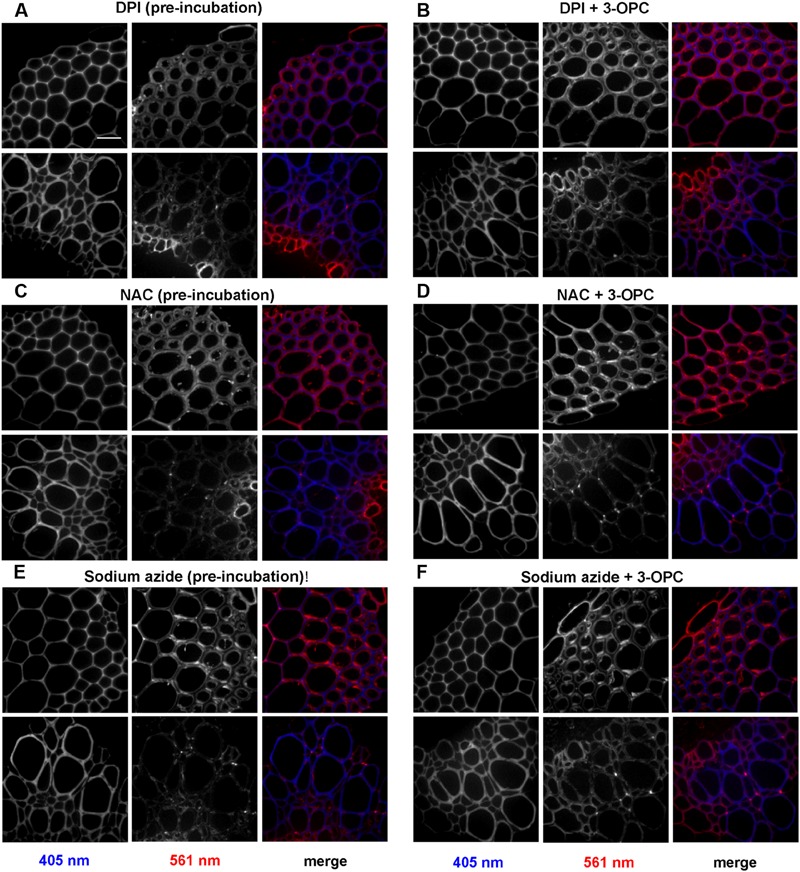
**Effect of inhibitors on incorporation patterns of 3-OPC in sections of 6-week-old *Arabidopsis* stem.** Inhibitors tested include: 15 μM DPI, an NADPH oxidase inhibitor **(A,B)**, 1 mM NAC, an antioxidant and ROS scavenger **(C,D)** and 1 mM sodium azide (NaN3), a peroxidase inhibitor **(E,F)**. **(A,C,E)** Click labeling (561 nm excitation) and autofluorescence (405 nm excitation) in sections treated with indicated inhibitors for 1 h, followed by 20 μM 3-OPC and 20 μM CA for 3 h, then labeled with Alexa 594-azide for 1 h, and washed with 96% ethanol for 1 h. **(B,D,F)** Click labeling (561 nm excitation) and autofluorescence (405 nm excitation) in sections treated with 20 μM 3-OPC and 20 μM CA along with the indicated inhibitors for 3 h, labeled with Alexa 594-azide for 1 h, and washed with 96% ethanol for 1 h. Both IFFs (top) and vascular bundles (bottom) are shown for each treatment. The colored images on the right column are merged for the 405 nm (blue) and 561 nm (red) channel. Images are contrast-enhanced maximum intensity projections of z series recorded using a 63× objective with a 561 nm laser at 5% power and 405 nm laser at 100% power, with 50 gain and 400 ms exposure time. Different ranges of brightness and contrast were used in order to observe any differences in incorporation patterns (scale bar, 20 μm). Three replicate experiments with at least three sections per experiment were imaged for each treatment.

**Figure 7** shows the effects of treating stem sections with inhibitors before or during 3-OPC incorporation on patterns and intensity of click labeling. Except for concurrent DPI treatment (*p* > 0.05, *t*-test), significant decreases in click labeling per unit area (*p* < 0.001, *t*-test) were measured for all treatments compared to controls (**Figure 7C**), indicating lower 3-OPC incorporation after treatment with all three inhibitors. This decrease occurred regardless of whether inhibitors were applied before or during 3-OPC incorporation. However, the magnitudes of decreases differed across treatments. When applied before 3-OPC incorporation, DPI greatly diminished 3-OPC incorporation, but when applied along with 3-OPC, DPI did not greatly reduce 3-OPC incorporation. Treatment with NAC before or during 3-OPC incubation reduced 3-OPC incorporation to similar, moderate degrees, whereas NaN_3_ treatment nearly abolished 3-OPC incorporation (**Figure 7C**). Higher magnification images (**Figure 8**) for IFFs and vascular bundles were collected for the samples described above, with brightness and contrast adjusted individually for each image to reveal 3-OPC incorporation patterns. There were no obvious differences in 3-OPC incorporation patterns when sections were treated with inhibitors. Although incorporation was diminished upon treatment with inhibitors, incorporation was still most evident in cell walls, as observed for 3-OPC incorporation at high concentrations without inhibitors (**Figure 2**). There were a few instances of incorporation at uneven intensities across the cell wall, which might correspond to locations where lignification was differentially inhibited.

## Discussion

The goal of this study was to examine the biochemical and developmental dependencies of lignification using the click-compatible monolignol analog, 3-OPC. We found that 3-OPC accurately mimics natural monolignol polymerization deposition. The patterns of stem section lignification by 3-OPC are consistent with natural patterns found at different developmental stages of the stems, with higher 3-OPC incorporation in tissues that are actively lignifying and lower 3-OPC incorporation in tissues that are already extensively lignified (**Figure 1A**). Autofluorescence in sections from older stem tissue showed large amounts of pre-existing lignin in IFFs and vascular bundles, which possibly precluded high levels of new lignification in these tissues. Thus, 3-OPC appears to be a specific marker for new lignification, given its minimal deposition on areas with large amounts of pre-existing natural lignin.

The lignification pattern exhibited by 3-OPC was not altered with addition of exogenous HRP (**Figure 1B**), but click labeling intensity per unit area increased in all tissues tested except 6-week-old top sections (Supplementary Figure S2). It is possible that increases in available lignification sites, but not radical-producing enzymes, as tissues matured accounts for these increases in labeling upon HRP addition. The H_2_O_2_ used by exogenous HRP in these experiments might be provided by NADPH oxidase enzymes that are present and active only in naturally lignifying cells, resulting in enhanced 3-OPC incorporation in only those cells. These results support the idea that localized lignin deposition depends on the localization of the enzymes required for lignification, including monolignol-oxidizing enzymes (peroxidases, laccases) as well as enzymes that generate H_2_O_2_ (e.g., NADPH oxidases) or O_2_ required for activity of oxidizing enzymes (Lee et al., 2013; Schuetz et al., 2014).

We found that supplying low concentrations of 3-OPC (without added CA) resulted in incorporation primarily at cell corners and middle lamellae (**Figure 2B**); however, at higher 3-OPC concentrations, incorporation occurred throughout the cell walls, with more intense labeling in secondary wall regions (**Figure 2**). The low levels of PI labeling we observed at cell corners and middle lamellae (**Figure 3**) might be attributable to pectin degradation as lignification proceeds, masking of pectin PI-binding sites by lignin or other wall components, or tight pectin crosslinking that precludes PI binding in these regions. It is possible that cell corners and middle lamellae contain higher concentrations of radical-generating enzymes, or enzymes with higher affinity or activity for monolignol radicalization, than secondary wall regions, and that monolignols therefore more readily polymerize in cell corners and middle lamellae, but that the cell corners and middle lamellae also contain limiting amounts of lignin initiation sites, and that once these sites are “saturated,” and/or when monolignol concentrations exceed a given concentration threshold, more lignin is deposited in cell wall regions. The different patterns of incorporation at low and high 3-OPC concentrations might be attributable to differences activity between laccases and peroxidases. Lee et al. (2013) found that Casparian strip lignification depends on peroxidases, whereas lignification in protoxylem tracheary elements appears to depend mostly on laccases (Zhao et al., 2013; Schuetz et al., 2014), though this cell specificity is not so clear cut, as both laccase mutants (Brown et al., 2005; Berthet et al., 2011) and peroxidase mutants (Herrero et al., 2013) display collapsed tracheary elements. Additionally, our results from experiments adding H_2_O_2_, HRP, or both (**Figure 6**) are consistent with the hypothesis that localization of H_2_O_2_-generating enzymes in lignifying cells is a key factor in determining where lignification occurs (Lee et al., 2013; Schuetz et al., 2014).

The concentration-dependent lignification patterns we observed do not appear to also be time-dependent, since the duration of incorporation did not appear to determine whether lignification occurred primarily in cell walls versus cell corners and middle lamellae (**Figures 4** and **5**). However, at high 3-OPC concentration, IFFs at 24 h time points did show concentrated spots of click labeling in walls that were not observed in 10 min samples where click labeling was more uniform (**Figure 4**). These spots might represent sites of higher enzyme activity or wall regions that become more amenable to 3-OPC deposition over the course of 24 h. In summary, no obvious changes in the localization patterns of 3-OPC deposition, besides concentrated spots of click labeling, were observed in stem sections over time, with only fluorescence intensities per unit area increasing up to 12 h and plateauing between 12 h and 24 h.

From our chemical inhibition experiments (**Figures 7** and **8**) provide further support for the idea that peroxidases, laccases, and H_2_O_2_-producing enzymes are important factors that control the incorporation of exogenously supplied monolignols, and that their inhibition results in lower lignification. The minor inhibition of 3-OPC incorporation upon concurrent treatment with DPI (**Figure 7**) suggests that residual H_2_O_2_ generated by NADPH oxidases might remain within stem sections even in the presence of DPI, whereas upon pre-incubation with DPI for 1 h, the washing step after inhibition might have removed significant amounts of stored H_2_O_2_, while not restoring NADPH oxidase activity. The incorporation of synthetic monolignols such as 3-OPC is likely to proceed through a free radical-coupling mechanism involving ROS, since treatment with the radical scavenger NAC resulted in lower 3-OPC incorporation (**Figure 7**). Laccases and peroxidases have non-redundant functions in vascular development in *Arabidopsis* (Zhao et al., 2013), even though both can polymerize monolignols *in vitro* (Sterjiades et al., 1992; Ranocha et al., 2002; Mechin et al., 2007). Treatment with NaN_3_, which irreversibly inhibits both peroxidases (Brill and Weinryb, 1967) and laccases (Patel et al., 2014; Johannes and Majcherczyk, 2000) treatment reduced 3-OPC incorporation most severely, implying that peroxidases and laccase might both be required for normal levels of synthetic lignification in *Arabidopsis* stem sections.

In summary, the click-compatible monolignol analog, 3-OPC, exhibits incorporation patterns that are consistent with natural lignification patterns in different developmental stages of *Arabidopsis* stems. Because 3-OPC mimics natural monolignols in its deposition patterns, it can serve as a probe to understand both the spatial/temporal dynamics and the molecular mechanisms underlying lignification. We demonstrate that localization of H_2_O_2_-generating enzymes is likely to help determine when and where lignification occurs, since when H_2_O_2_ and peroxidase are exogenously supplied, even non-lignifying cells can be artificially lignified. Inhibitors of factors known to be involved in lignification reduced 3-OPC incorporation in *Arabidopsis* stem sections, confirming that 3-OPC incorporation proceeds through an enzyme-generated free radical-polymerization mechanism involving ROS and is therefore a reliable monolignol analog. Surprisingly, modulating 3-OPC concentration affected where lignification occurs, with lower 3-OPC concentrations enabling lignification primarily in cell corners and middle lamellae and higher concentrations enabling lignification primarily in cell walls. Therefore, a complex interplay between monolignol concentration, enzyme localization and activity, and lignin nucleation site density and affinity might be responsible for the subcellular patterns of lignification we and others have observed. Incorporation of 3-OPC at concentrations below 0.2 μM might closely mimic the initial stage of the lignification process, which begins in the middle lamellae and cell corners and proceeds through the cell wall. Plant cells might thus be able to control where and when lignification occurs by modulating monolignol production or transport localization and rates. Future application of 3-OPC and other molecular probes for lignification should prove to be useful for further dissection of the cellular and molecular factors that control lignification, unraveling this complex process to help us understand how lignocellulosic biomass is constructed and improving our ability to use this renewable resource to benefit human society.

## Author Contributions

JP, SK, TR, YZ, DC, and CA designed the experiments; JP, SK, and YZ performed the experiments; JP, SK, TR, YZ, DC, and CA analyzed the data; JP, SK, TR, YZ, DC, and CA wrote the paper.

## Conflict of Interest Statement

The authors declare that the research was conducted in the absence of any commercial or financial relationships that could be construed as a potential conflict of interest.
