# Supplementary material for: Investigating Biochemical and Developmental Dependencies of Lignification with a Click-Compatible Monolignol Analog in Arabidopsis thaliana Stems
Source: Front Plant Sci. 2016 Aug 31;7:1309. doi: 10.3389/fpls.2016.01309 (PMC5005335; doi:10.3389/fpls.2016.01309)
Supplement: Supplementary file 1 [file Presentation_1.PDF]

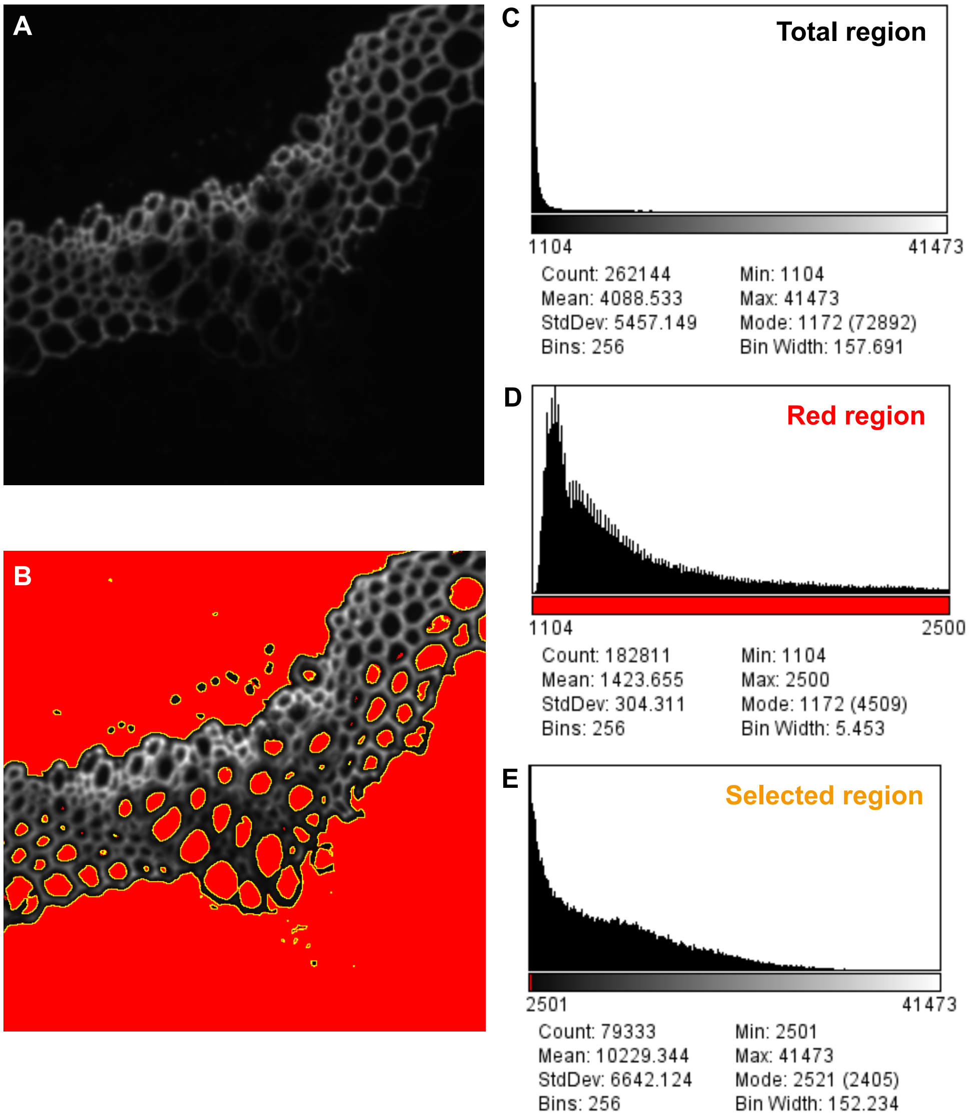

Figure S1. Selection of lignified area for quantification analysis of fluorescence intensities per unit area for click labeling-associated fluorescence (561 nm excitation) as well as autofluorescence (405 nm excitation) in stem sections incorporated with 3-OPC. (A) An example image for which the click-labeling-associated fluorescence intensity of the area where 3-OPC has been incorporated needs to be quantified. (B) Same image where low intensity regions with gray values below 2500 have been thresholded out (shown in red) and the regions with gray values 2501 and above have been selected. (C) Histogram showing gray values for the entire image A. (D) Histogram showing gray values for the regions in red thresholded out in image B. (E) Histogram showing gray values for the selected "lignified" region in image B. In order to calculate the click-labeling associated fluorescence intensity per unit area for this image, the fluorescence intensity of the selected region is determined and divided by its selected area in pixels. The same threshold value (2500, in this example) is then used to quantify fluorescence intensities of all images that needed to be compared in a particular quantification analysis.

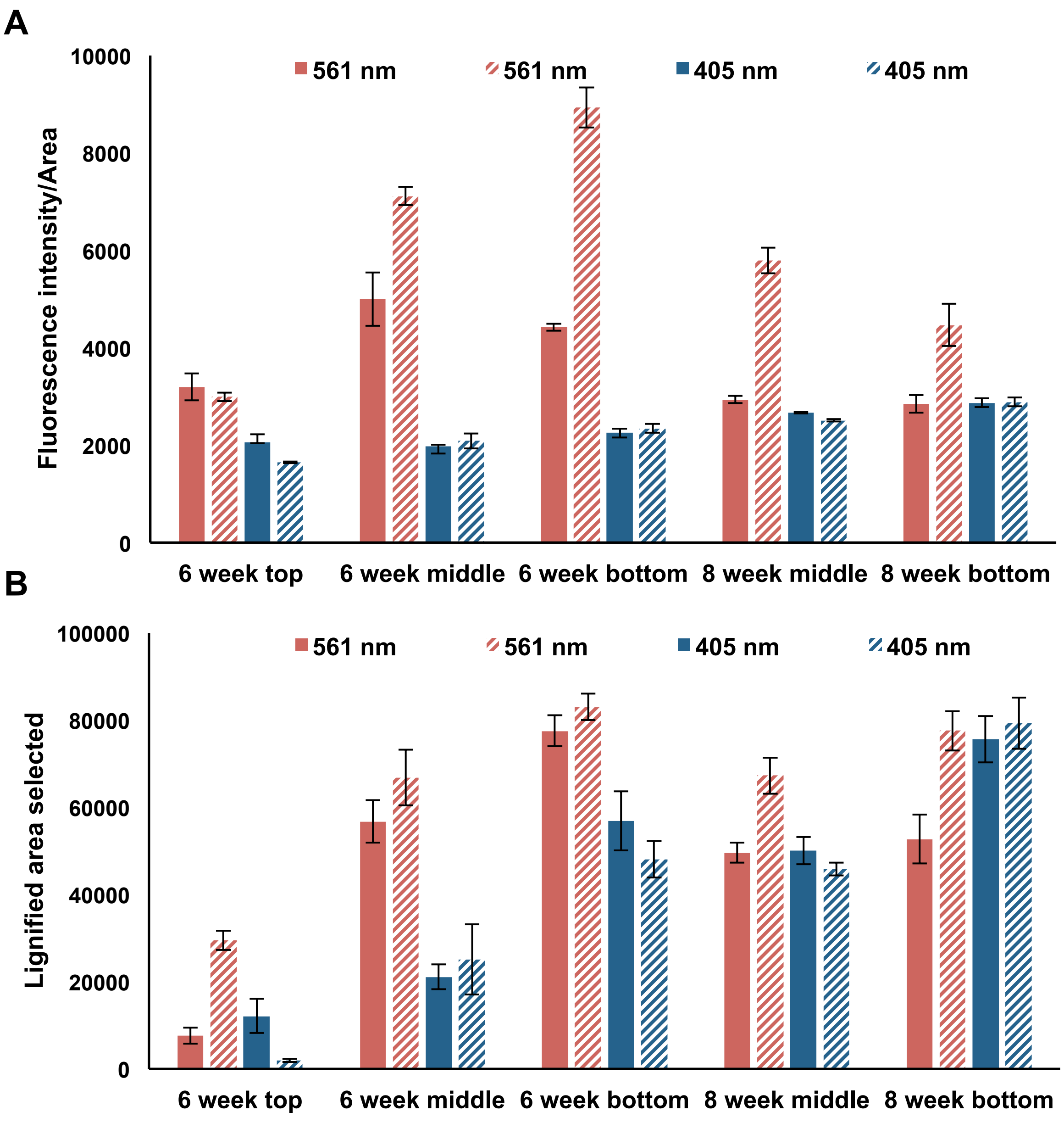

Figure S2. Quantification analysis of Figure 1. (A) Quantification of fluorescence intensities per unit area for click labeling-associated fluorescence (561 nm excitation, red) and autofluorescence (405 nm excitation, blue) in 40 µm-thick sections of top, middle and bottom portions of 6-week-old and middle and bottom portions of 8 week old *Arabidopsis* stem, treated with 20 µM 3-OPC + 20 µM CA without exogenous HRP (solid) and with exogenous HRP (striped) for 3 h, labeled with Alexa 594-azide for 1 h, and washed with 96% ethanol for 1 h. (B) Quantification of lignified (thresholded) area selected for the 561 nm and 405 nm channels in the fluorescence quantification analysis for each of the samples described above. Data were averaged from three replicate experiments with three sections images for each treatment; error bars indicate standard error.

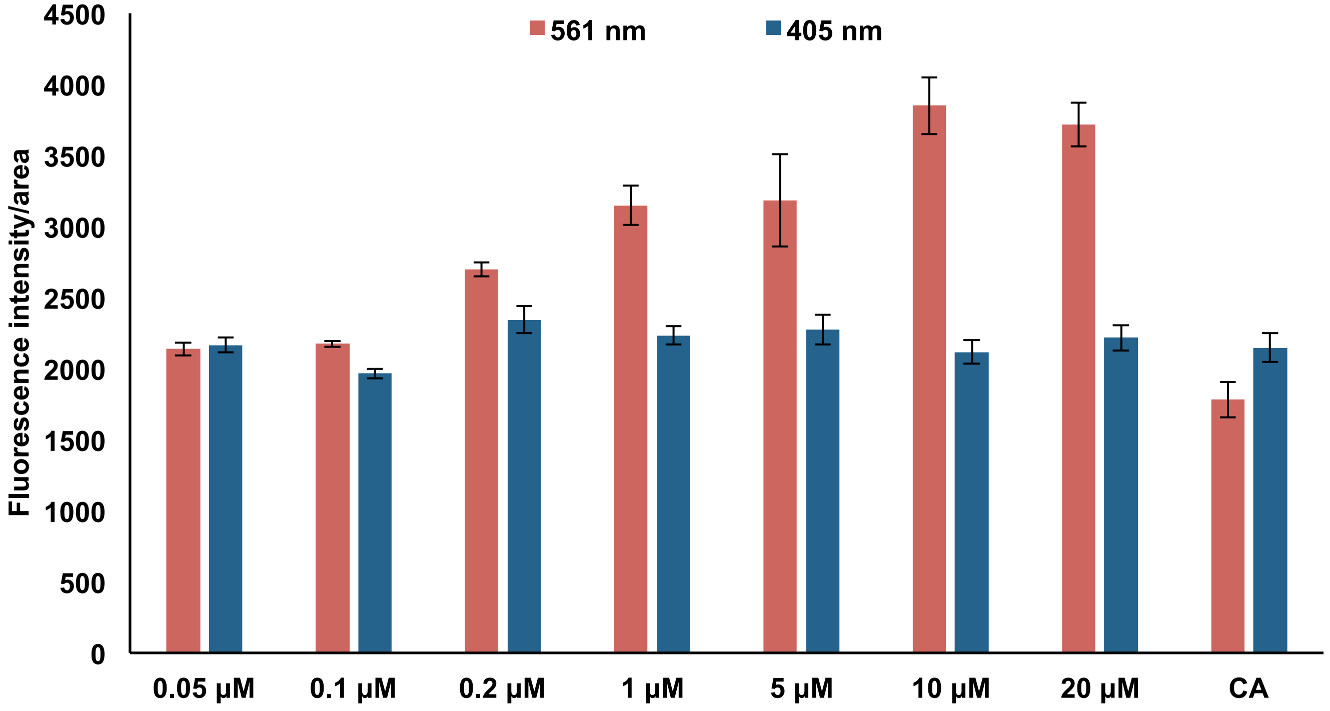

Figure S3. Quantification analysis of Figure 2. Quantification of fluorescence intensities per unit area for click labeling-associated fluorescence (561 nm excitation, red) and autofluorescence (405 nm excitation, blue) in sections treated with 0.05 μM, 0.1 μM, 0.2 μM, 1 μM, 5 μM, 10 μM and 20 μM 3-OPC and control sections treated with 20 μM CA for 3 h, labeled with Alexa 594-azide for 1 h, and washed with 96% ethanol for 1 h. Data were averaged from three replicate experiments with three sections images for each treatment; error bars indicate standard error.

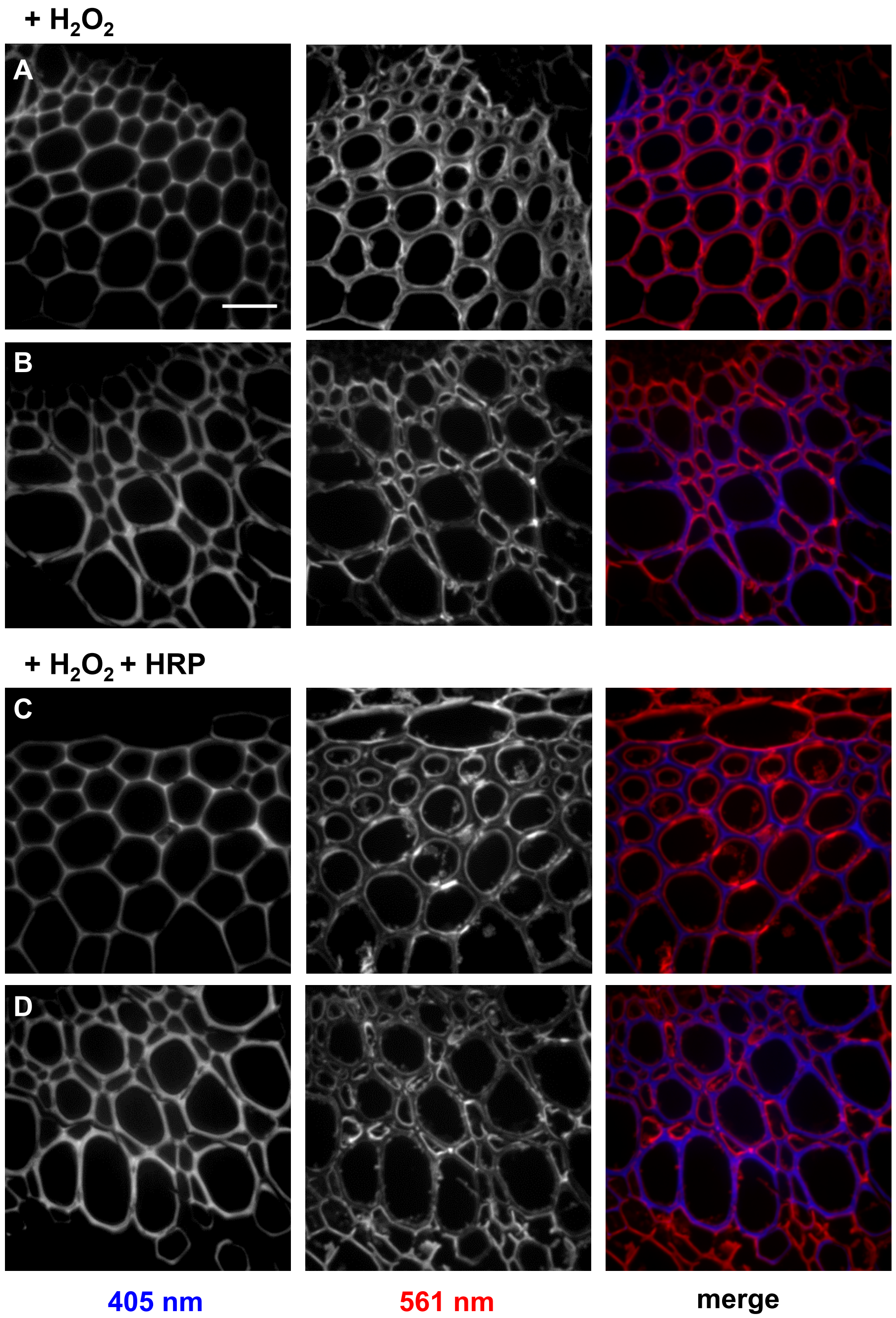

Figure S4. Supplemental figure for figure 6. High magnification images for effect of addition of peroxide on lignification patterns. Patterns of 3-OPC incorporation in presence of exogenous hydrogen peroxide in 40 μm-thick sections of 6-week-old *Arabidopsis* stem. Autofluorescence (405 nm excitation) and click labeling (561 nm excitation) in sections treated with 20 μM 3-OPC + 20 μM CA in (A, B) presence of 0.001% H_2O_2 and (C, D) presence of 0.001% H_2O_2 as well as exogenous HRP, for 3 h, labeled with Alexa 594-azide for 1 h, and washed with 96% ethanol for 1 h. A and C show IFFs and B and D show vascular bundles. The colored images on the right column are merged for the 405 nm (blue) and 561 nm (red) channel. Images are contrast-enhanced maximum intensity projections of z series recorded with a spinning disk fluorescence confocal microscope recorded using a 63X objective with a 561 nm laser at 5% power and a 405 nm laser at 100% power, with 50 gain and 400 msec exposure time. Different ranges of brightness and contrast were used in order to observe any differences in incorporation patterns (scale bar, 20 μm).
